# Construction and Use of Active Thermal Markers

**DOI:** 10.3390/s26216981

**Published:** 2026-11-04

**Authors:** Paulina Lewińska, Kamil Maciuk, Łukasz Więckowski, Paweł Postek, Sorin Nistor, Łukasz Borowski

**Affiliations:** 1 Faculty of Geo-Data Science, Geodesy, and Environmental Engineering, AGH University of Krakow, 30-059 Krakow, Poland; 2 HOGERBOX Sp. z o.o., ul. Lipowa 3, 30-702 Kraków, Poland; 3 Faculty of Production Engineering, University of Life Sciences in Lublin, 20-612 Lublin, Poland; 4 Faculty of Construction, Cadastre and Architecture, University of Oradea, 410058 Oradea, Romania; 5 Institute of Geography and Spatial Studies, University of the National Education Commission, 30-084 Krakow, Poland

**Keywords:** thermal point cloud, infrared thermograph, terrestrial laser scanning, coal spoil tips

## Abstract

Thermal monitoring of complex topographic objects requires accurate integration of infrared data with three-dimensional spatial information. However, registering thermal imagery with point clouds remains challenging due to limited spatial resolution, low contrast, and the lack of stable reference features in thermal images. This study presents a methodology for generating a thermal point cloud using active thermal markers as control points. The developed markers are based on temperature-controlled Peltier modules, providing stable and detectable thermal signatures independent of the natural temperature distribution of the investigated surface. Laboratory and field experiments evaluate marker stability, detection capability, and applicability for integration with Terrestrial Laser Scanning (TLS). An automated procedure was developed to identify thermal markers and transfer temperature information from infrared images to point clouds using a Direct Linear Transformation approach. The proposed workflow was tested on a coal spoil tip, where a combined spatially referenced model was made. The results confirmed that active thermal markers enable reliable thermal image registration and allow temperature values to be assigned to three-dimensional terrain models. The proposed approach can support monitoring of mining areas, detection of potential zones of heat accumulation, and early identification of areas at risk of spontaneous combustion.

## 1. Introduction

Infrared (IR) thermography, often referred to as thermal imaging, is a method used to detect and visualise infrared radiation emitted by objects, which can be later calculated and interpreted as a temperature value on the object’s surface or near the surface [1,2]. This technology enables the observation of temperature variations across a surface, which can be valuable for various applications, including identifying differences in material properties, moisture content, or structural conditions. “Short-range” infrared thermography typically refers to using thermal imaging cameras at close range to the target object. In this context, short-range thermography can provide high-resolution thermal images, allowing for detailed analysis of thermal anomalies on the object’s surface.

The Terrestrial Laser Scanning (TLS) process generates a point cloud representing the surface geometry of scanned objects. A stationary laser scanner operates as follows: the scanner head (mirror) rotates in a preprogrammed sequence, emitting a laser beam while recording the corresponding angular information. When the beam returns, the distance to the target is computed either by measuring the time interval between emission and reception (impulse scanners) or by analysing the phase shift in the returning signal (phase scanners). The resulting point cloud is a set of XYZ coordinates that describe the object’s surface. In addition to spatial information, TLS also provides auxiliary data, including intensity values (I), which represent the strength of the returned signal, and RGB values, which represent the colour of measured points [3,4]. Apart from the obvious advantages of terrestrial laser scanning, such as fast, precise data acquisition and high spatial detail, some disadvantages should be considered when choosing this measurement technology. One of them is the large volume of resulting data and the associated problems at the point cloud processing and storage stages, too [5].

Integrating three-dimensional point clouds with infrared (IR) data provides a comprehensive representation of both the geometric structure and thermal characteristics of an observed scene. While point clouds accurately describe the spatial geometry, shape, and surface morphology of objects, thermal imagery provides radiometric information on surface temperature and heat distribution. Combining these complementary datasets enables the identification of features and processes that neither data source can detect alone. As a result, thermal point clouds have the potential to become a valuable tool in environmental monitoring, infrastructure inspection, precision agriculture, and disaster assessment, where both spatial and thermal information are essential for accurate analysis and decision-making.

In the context of monitoring natural and topographic objects, integrating point clouds with IR data offers significant advantages for analysing terrain, vegetation, water bodies, and geological features. Three-dimensional thermal models help detect thermal anomalies associated with groundwater discharge, soil moisture variability, geothermal activity, erosion processes, or vegetation stress, while preserving the precise spatial relationships between these phenomena and the surrounding terrain. Accurate topographic information also enables thermal measurements to be interpreted relative to surface orientation, slope, elevation, and object geometry, reducing ambiguities caused by viewing geometry or terrain occlusions.

In this study, we present a method for directly linking IR images to TLS or other point clouds using specifically designed thermal markers. The contributions of this work are as follows:

- Designing and testing active thermal markers;
- Creating an algorithm for semi-automatic creation of a thermal point cloud (TCP).

We present a semi-automatic method for creating IR point clouds. Additionally, we have integrated infrared thermography and terrestrial laser scanning to improve interpretation of the structure of the deposit material at coal spoil tips. In this paper, active thermal markers are artificial control targets equipped with an internal heating/cooling system that actively maintains a predefined surface temperature, creating a stable thermal contrast with the surrounding environment. This controlled thermal signature enables reliable, semi-automatic detection in infrared imagery under varying environmental conditions. In contrast, passive thermal markers lack an internal temperature-control system and rely solely on the natural thermal properties (e.g., emissivity, reflectivity, or thermal inertia) of their constituent materials to generate contrast in thermal images. Consequently, their visibility depends on ambient conditions and is generally less stable than that of active markers.

## 2. Related Work

In recent years, integrated measurement methods have developed rapidly, utilising various data and observations such as LiDAR, InSAR, GNSS, TLS, and thermal imaging [6,7,8]. Despite growing interest in multi-sensor data fusion, integrating LiDAR and thermal imagery remains challenging because of several technical limitations. Unlike conventional LiDAR–RGB systems, LiDAR–thermal fusion requires accurate sensor-to-sensor calibration, data synchronisation, and precise mapping of thermal information into three-dimensional space. These challenges are further exacerbated by thermal cameras’ inherent characteristics, including lower spatial resolution, limited image contrast, and reduced thermal texture, which hinder reliable feature extraction, multimodal correspondence matching, and accurate data alignment. Additional difficulties arise from differences in sensor fields of view and the heterogeneous nature of the acquired data. Although numerous calibration methods have been developed for LiDAR–RGB systems, relatively few studies address the practical integration of LiDAR and radiometric thermal cameras under real measurement conditions. Moreover, many existing approaches rely on complex optimisation algorithms or deep learning frameworks that require extensive training datasets and significant computational resources.

### 2.1. Integrating IR and Point Cloud Data

Integration of point clouds with infrared information can be done in various ways, but it can be challenging [9]. There are applications that allow installing IR cameras on top of TLS units and then cover the point cloud with pre-calibrated panoramas from those cameras. A limitation of this method involves range. Usually, the range of the TLS is much greater than the usable range of IR cameras (due to both pixel size and environmental conditions that affect the quality of thermal readings). This cannot always be mitigated by adding additional scan stations for two reasons. First, it is not always possible to access the object to add more close-range scan stations, and second, even if placing extra stations is possible, the measurement might not be valid due to changes in environmental conditions that affect the IR reading. Another option is to create an IR point cloud using a Structure-from-Motion (SfM) algorithm [10]. This can be done if a sufficiently high-quality camera is available; however, this kind of point cloud generally has lower precision than a TLS point cloud. Additionally, due to IR camera limitations, including low pixel counts in most affordable models, these models are often not detailed enough [11]. For larger areas, integration of satellite-based IR images with point clouds is possible [12]. However, the pixel size of those images is large, so the resulting models lack detail. Additionally, those models require extensive interpretation, as IR data is highly influenced by weather conditions. Thus, if not handled correctly, the information obtained may be misleading; also, nominal temperatures are hard to extract, so the analysis relies on temperature differences.

The most common problem is how to connect IR 2D data to 3D data. For RGB data, this can be done easily with predefined points, Ground Control Points (GCPs), or easily defined object elements such as window corners or building edges. However, this becomes more difficult with topographic objects such as spoil tips or landslide areas since they lack those characteristic elements. What is more, even if they exist, they are not visible in IR. Additionally, traditional man-made GCPs are often not visible in IR; thus, there is a need to design custom elements that can serve as GCPs or points in IR images.

### 2.2. Strategies for Integrating IR Data with Point Clouds

In contrast to conventional RGB photogrammetry, where surveyed ground control points (GCPs) are commonly employed for image orientation and georeferencing [13], most LiDAR/SfM–thermal fusion studies do not rely on traditional GCPs for data registration. Many methods avoid physical targets altogether by exploiting LiDAR intensity images to establish correspondences, extracting manually or semi-automatically identified multimodal key points, or matching geometric features such as corners, planes, and edges between the point cloud and thermal images [14,15]. While these methods have demonstrated promising results, they often require carefully controlled acquisition conditions, specialised calibration targets, or computationally demanding registration procedures, highlighting the continued need for simple and robust approaches for integrating thermal imagery with three-dimensional point clouds [16].

Another approach relies on calibration strategies specifically designed to address the differing imaging characteristics of LiDAR and thermal sensors. Unlike visible-light cameras, thermal cameras require calibration targets that generate sufficient radiometric contrast for reliable feature detection. Consequently, researchers have proposed various target designs, including checkerboard patterns made from materials with contrasting emissivities, metal–paint composite targets, and actively heated or cooled calibration plates [17]. Passive targets exploit emissivity differences between adjacent materials, whereas active targets use electrical heaters or thermoelectric (Peltier) elements to generate stable thermal patterns, thereby improving feature visibility and calibration accuracy [18]. Recent developments have also introduced inexpensive composite calibration boards [19] and thermally enhanced ChArUco targets that enable accurate corner detection under varying environmental conditions [20].

Although these calibration strategies have significantly improved the geometric calibration of infrared cameras and RGB–thermal camera systems, their application to LiDAR–thermal data fusion remains limited. Most existing targets have been developed for camera calibration rather than for registering thermal imagery with three-dimensional point clouds, and only a few studies consider targets simultaneously detectable by both LiDAR and thermal sensors. Additionally, very little work uses thermal targets in fieldwork conditions. Existing papers propose either very large units that are used for general calibration of images or very specific applications [21,22]. Consequently, developing robust thermal control points suitable for multimodal registration and thermal point cloud generation remains an open research challenge.

## 3. Materials and Methods—Designing Thermal Markers and an Algorithm for the Creation of IR Point Clouds

### 3.1. General Idea Behind Thermal Markers

The active thermal marker concept was based on developing a low-cost yet precise device for thermographic measurements, providing a clearly visible and easily identifiable target in thermal images. The design objectives were to maintain a predefined temperature with minimal fluctuations and to achieve the highest possible temperature uniformity across the Peltier module, which constituted the active component of the marker. Under controlled laboratory conditions, at an ambient temperature of approximately 20 °C, the target temperature was expected to remain stable within ±0.5–2.0 °C. To meet these requirements, the marker was constructed from a 100 × 100 mm aluminium plate coupled with a suitably selected thermoelectric (Peltier) heating/cooling module [23]. This size was chosen to ensure reliable detection in thermal images acquired with a typical 320 × 240-pixel infrared camera at distances of up to approximately 30 m. Such cameras are widely used for general-purpose thermographic applications. Larger targets would significantly increase manufacturing costs and reduce portability, while the overall marker dimensions were also constrained by the heating/cooling module’s size, power requirements, and power supply.

### 3.2. Construction of the Thermal Markers

**Figure 1:**
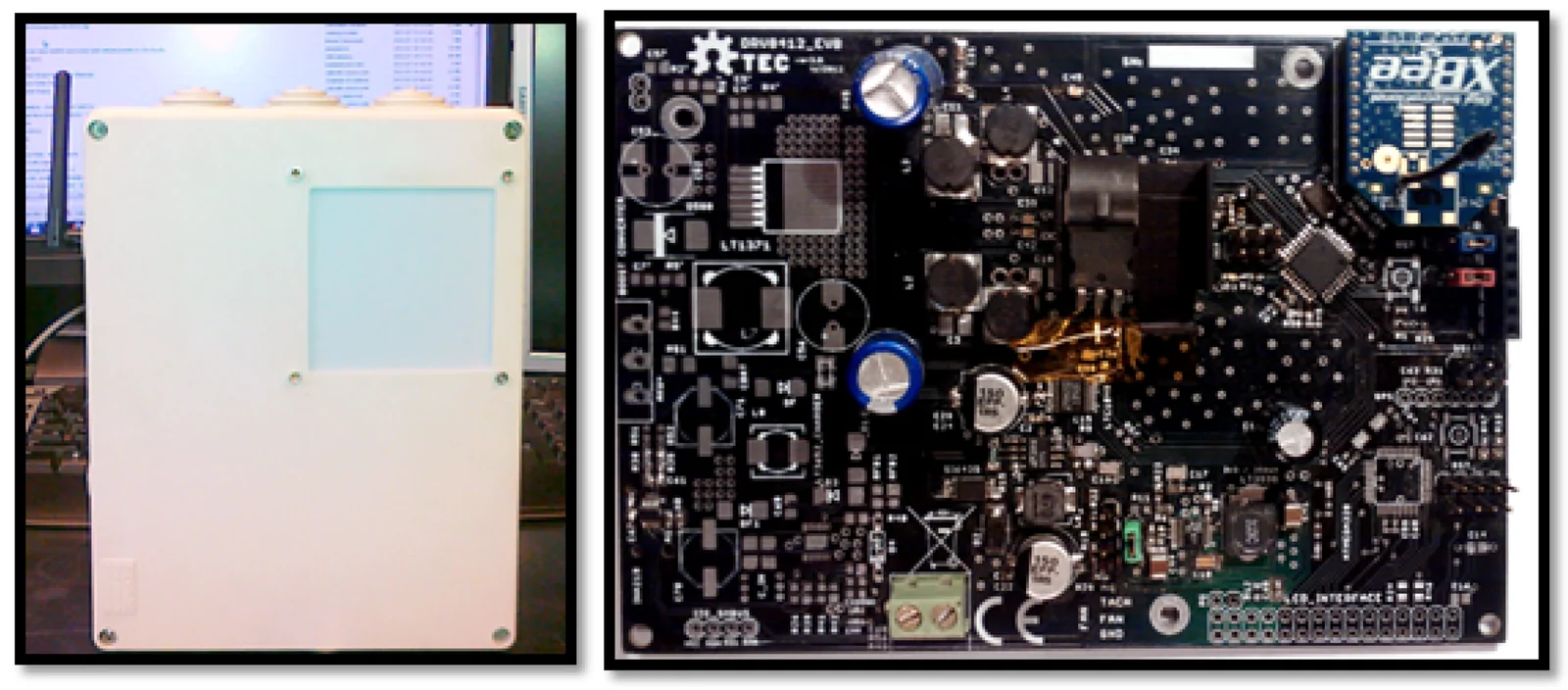
(**Left**)—active thermal marker design. (**Right**)—used PCB design.

The thermal marker was designed around a low-power microcontroller that handled temperature regulation and environmental data acquisition (Figure 1). In addition to maintaining a stable target temperature, each marker recorded ambient temperature, relative humidity, and illumination. The system was powered by rechargeable batteries, providing 2–4 h of continuous operation, and was intended to operate as part of a wireless sensor network consisting of multiple identical markers managed by a central control unit. The integrated electronics were implemented on a single printed circuit board, resulting in a compact and portable design. Particular emphasis was placed on energy efficiency through continuous monitoring of battery status and power consumption, enabling real-time estimation of the remaining operating time for each marker.

Based on the design requirements, the thermal marker was constructed using a Peltier module with a maximum power of 50 W, mounted on an aluminium plate measuring 100 × 100 × 10 mm. A Peltier module is a semiconductor device composed of two thin ceramic plates connected by a series of semiconductor elements that enable the Peltier effect. This effect occurs when an electric current passes through the junction between two different semiconductor materials, causing one side of the junction to absorb heat while the other releases it, resulting in a temperature difference between the two sides of the module. Depending on the direction of current flow, one side of the plate is heated while the other is cooled [23,24]. By controlling the magnitude and polarity of the supplied current, the heat transfer can be adjusted, allowing precise regulation of the surface temperature of the marker.

**Figure 2:**
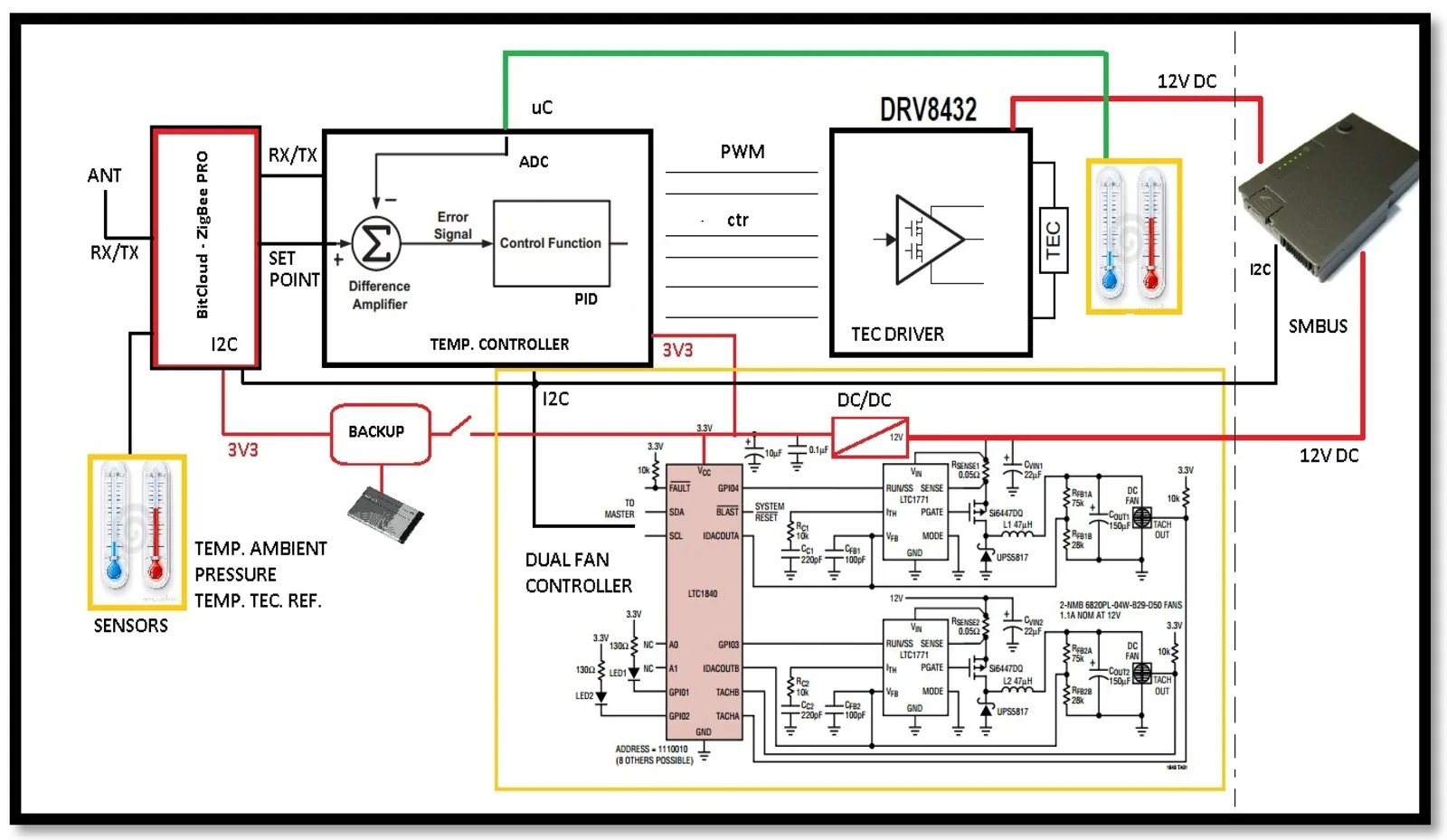
Thermal markers operation diagram.

The Peltier module was driven by a DRV8412 integrated dual full-bridge power driver (Texas Instruments, Dallas, TX, USA) [25], providing a high-efficiency power stage (up to 97%) and supporting pulse-width modulation (PWM) control at switching frequencies of up to 500 kHz. The output power delivered to the Peltier module was regulated using PWM generated by the microcontroller, while a digital PID controller continuously adjusted the duty cycle to maintain the desired surface temperature [25]. Within the control loop, the predefined target temperature was continuously compared with the actual temperature of the marker plate, measured using an integrated temperature sensor. The temperature difference was compensated using a digital proportional–integral–derivative (PID) controller implemented in the microcontroller. To improve heat transfer between the two sides of the Peltier module, the inner side of the thermal marker plate was equipped with a heat sink and a fan with an integrated speed controller. In addition to continuous temperature stabilisation, the microprocessor system collected data from digital environmental sensors connected to the communication bus (Figure 2). The system incorporated a Sensirion SHT12 temperature and humidity sensor and a digital ambient light sensor [26]. Communication with the host unit was provided by a ZigBee-based radio transceiver module [27]. A dedicated communication protocol was developed to enable reliable control and management of data obtained from multiple identical devices operating within the network. Each marker was powered by a 14.8 V lithium-ion battery pack with a 5 Ah capacity and an integrated battery performance monitoring system. The load current and supply voltage were continuously measured, allowing real-time calculation of power consumption and energy usage. This provided information on the current energy status of each thermal reference marker and enabled estimation of the remaining operating time. The maximum operating duration depended on the difference between the predefined marker temperature and the ambient environmental temperature. Practical experiments demonstrated stable operation for at least 2 h under typical conditions, with an average power consumption corresponding to approximately 50% of the device’s maximum power capacity.

**Figure 3:**
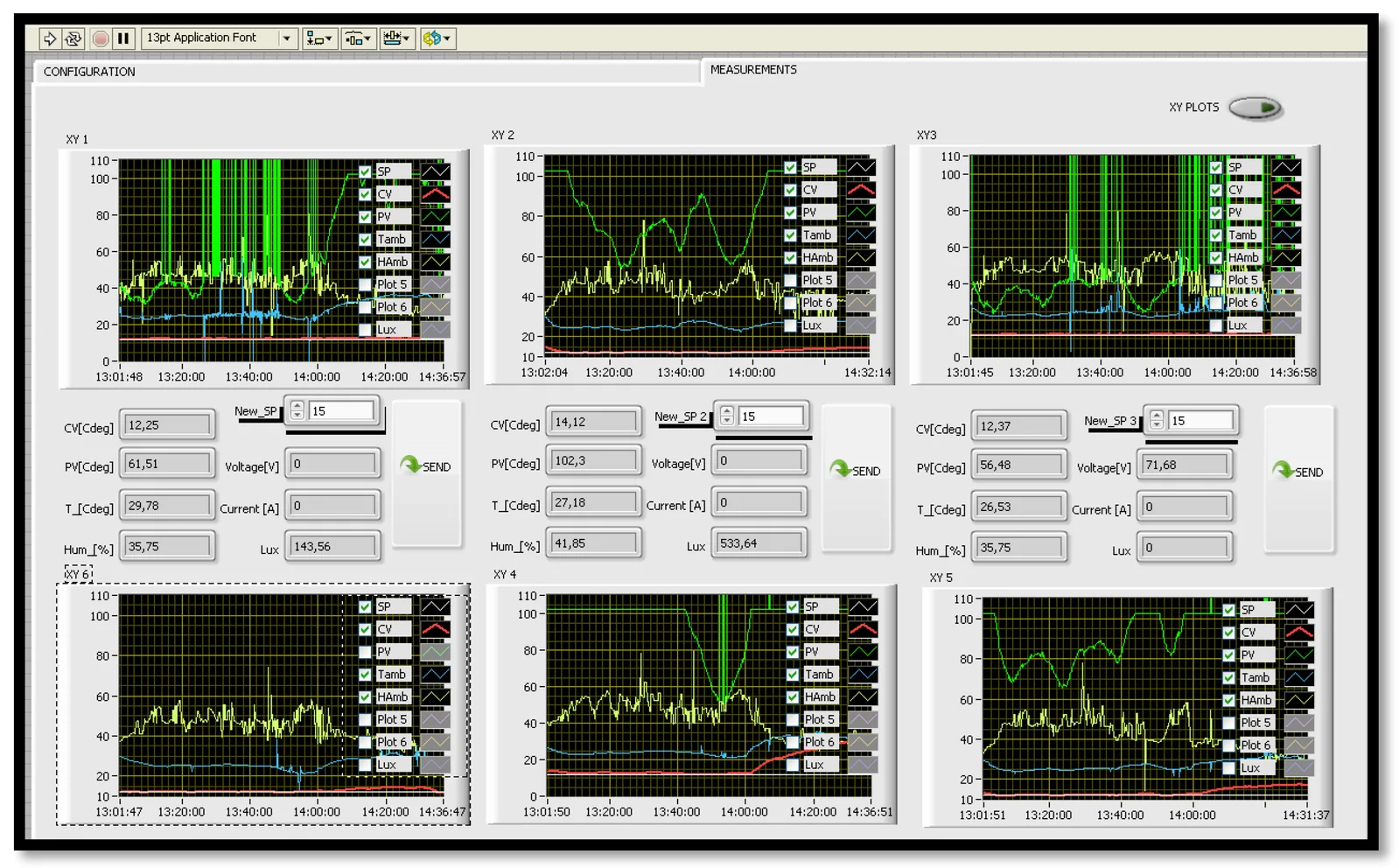
Interface of the software controlling the operation of the thermal markers; each application window corresponds to an individual marker.

Additionally, a dedicated application was developed in the LabVIEW environment to control and monitor the thermal markers. The application enables the user to set the target temperature to be maintained by the Peltier module (Figure 3). At one-second intervals, it receives measurements of the marker plate temperature, as well as battery consumption and illuminance at the marker surface, both expressed in lux. All collected data are automatically recorded in a text file for further analysis.

### 3.3. Direct Linear Transformation (DLT)

To integrate the point cloud with thermal imagery, it was necessary to establish the relationship between object-space coordinates and image-space coordinates of each thermogram. For this purpose, the Direct Linear Transformation (DLT) algorithm was adopted, as it provides a well-established solution for transforming three-dimensional object coordinates into two-dimensional image coordinates without requiring explicit knowledge of the camera’s interior or exterior orientation parameters [28]. The DLT algorithm describes the projective relationship between a point in object space, (*XYZ*), and its corresponding image coordinates, (*xy*), by means of eleven transformation parameters:

(1) $$\left\{\begin{array}{c} x=\frac{L_{1X}+L_{2Y}+L_{3Z}+L_{4}}{L_{9X}+L_{10Y}+L_{11Z}+1}\\ y=\frac{L_{5X}+L_{6Y}+L_{7Z}+L_{8}}{L_{9X}+L_{10Y}+L_{11Z}+1} \end{array}\right.$$

where *L*_1_, …, *L*_11_ are the DLT coefficients determined from a set of homologous control points with known coordinates in both object and image space *x_i_*, *y_i_* are coordinates of point *i* from O, *X_i_*, *Y_i_*, and *Z_i_* are coordinates of point *i* from T.

A minimum of six non-coplanar control points is required to determine the transformation parameters. In practice, a larger number of control points is recommended, allowing the coefficients to be estimated using a least-squares adjustment, thereby improving the robustness and accuracy of the transformation [28]. Equally important is the spatial distribution of the control points, which should surround the study area and be evenly distributed to minimise extrapolation errors and maximise transformation accuracy [29].

In this study, the DLT algorithm transformed terrestrial laser scanning (TLS) point cloud coordinates into each thermogram’s image coordinate system. Once the transformation parameters were estimated, the algorithm projected each point in the point cloud onto the corresponding thermal image, enabling the assignment of temperature values to individual points. The algorithm was implemented in MATLAB R2024b (24.2) as a dedicated routine to compute the DLT coefficients from user-defined image- and object-space control points.

### 3.4. Semi-Automatic Detection of Thermal Markers

Thermal images were exported as comma-separated value (CSV) files containing the temperature recorded for each image pixel. The data were imported into MATLAB, where a custom processing routine was developed to semi-automatically detect the active thermal markers and determine their image coordinates. The import procedure was designed to handle occasional formatting inconsistencies in the exported files, ensuring robust processing of large thermal image datasets.

Marker detection was based primarily on temperature rather than geometric shape, as the square geometry of the active marker was not always preserved in the thermal images due to image resolution, viewing distance, and thermal diffusion. The algorithm first established a local image coordinate system and then applied a temperature threshold corresponding to the expected operating temperature of the thermal markers. During the experiments, the marker surface temperature typically varied by approximately 2–3 °C; therefore, the threshold interval was selected accordingly and could be adjusted to account for different environmental conditions or measurement uncertainties.

Pixels falling within the specified temperature range were converted into a binary image, where candidate marker pixels were assigned a value of one and all remaining pixels a value of zero. Connected-component analysis using an eight-neighbourhood connectivity criterion was then performed to group adjacent pixels belonging to the same thermal target. Since individual hot pixels could result from sensor noise or local temperature anomalies, isolated pixels were rejected, and only connected regions exceeding a predefined minimum size (typically five pixels) were accepted as valid markers. The image coordinates of each detected marker were subsequently determined by calculating the centroid of the corresponding connected region using the arithmetic mean of the pixel coordinates.

**Figure 4:**
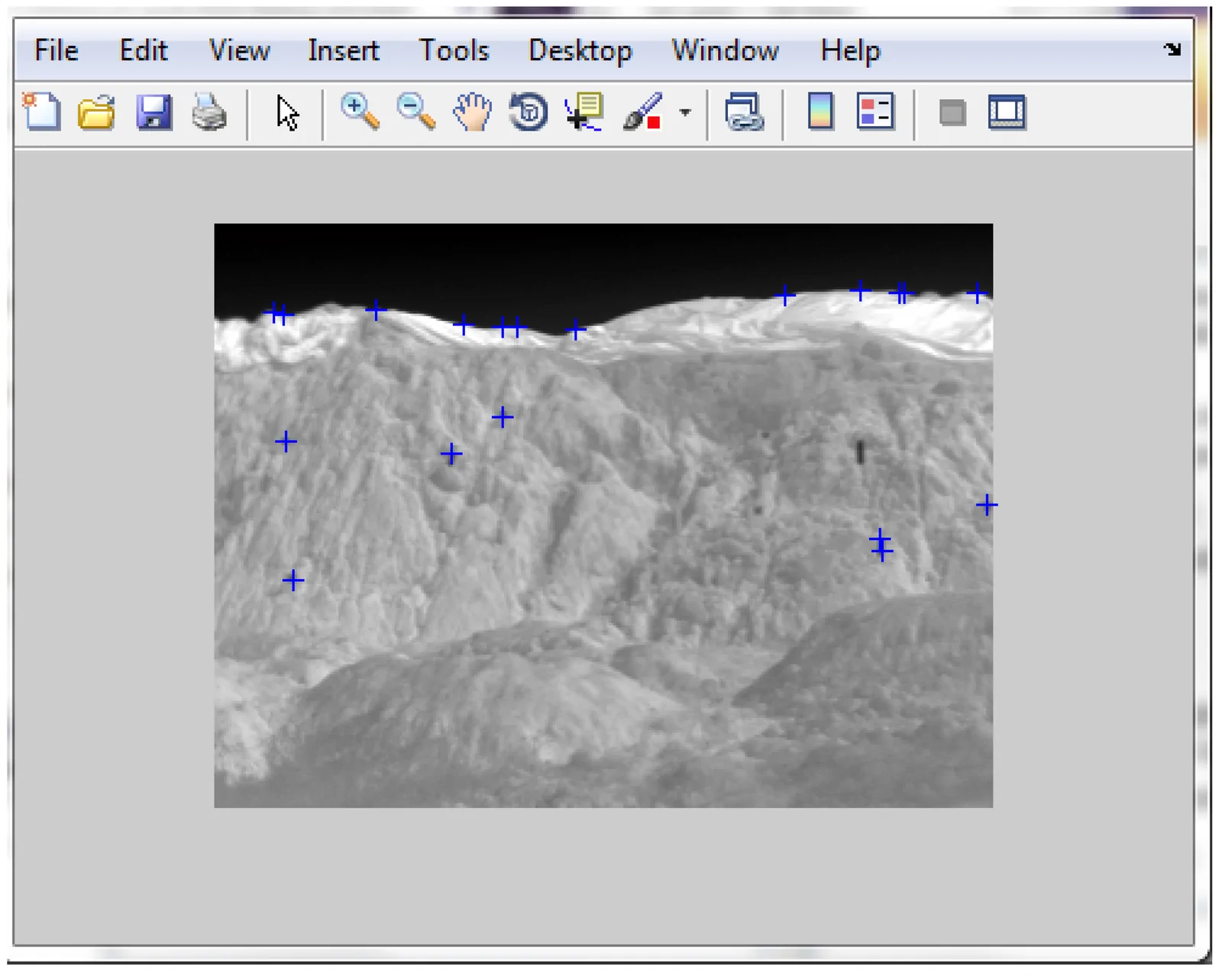
Thermogram of the test object showing the detected thermal markers (symbolised by blue crosses) and surface points with temperatures corresponding to the markers.

The detected marker positions were finally overlaid on the thermal image to allow visual inspection and, if necessary, manual removal of incorrectly identified targets (Figure 4). The resulting image coordinates served as homologous control points for the Direct Linear Transformation (DLT), enabling accurate registration of thermal images with the terrestrial laser scanning (TLS) point cloud and subsequent assignment of temperature values to the three-dimensional dataset.

The method is considered semi-automatic because it still requires human intervention to check the automatic detection results and to label/add the marker coordinates in the correct sequence. During the test survey, we numbered the markers and provided a sketch showing their coordinates. Then, we entered these coordinates into the MATLAB script, assigning the lowest number to the ‘leftmost’ marker in each image, and so on. However, we could perform the procedure only after inspecting the image and removing all false positives (pixels incorrectly detected as markers).

To evaluate the proposed method, we used a metric analysis. The images were separated into two groups: the first contained only the object, while the second also included the sky (e.g., Figure 4). In the first group, the number of false positives was significantly lower than in the second group. This was because temperature values tend to become more similar at the boundary between areas with large temperature differences. Initially, we attempted to mask the sky in various ways, but these proved ineffective, as the images still required visual (manual) inspection, just at a different stage of processing.

For all images, the detection rate of the active markers was 91%, calculated as the proportion of actual active markers that were correctly detected. However, in some cases, the same marker was detected twice, requiring the removal of one of the duplicate detections (Figure 4). Duplicate detections occurred in 13% of cases. For the first image type, the false-positive detection proportion was very low, at 5% of all detections, representing objects incorrectly identified as active markers. In the second image type, the false-positive detection proportion was 70% of all detections, with almost all false positives occurring along the skyline edge. These statistics are based on 38 images collected during the experiment described in Section 4.2, comprising 15 images of the second type and 23 of the first type. The results obtained in the experiments described in Section 5.2 were very similar.

## 4. Testing of Proposed Method

In the following section, we will describe field tests that allowed us to validate the quality of both thermal markers and the proposed processing algorithm.

### 4.1. Laboratory Evaluation of Active Thermal Markers

We evaluated the developed active thermal markers under laboratory conditions to verify their radiometric properties, temperature stability, operational performance, and suitability as thermal control points for image registration. The experiments focused on determining the emissivity of the marker surface, assessing the stability of the temperature control system under different environmental conditions, and investigating the influence of acquisition geometry on the visibility and apparent temperature of the markers.

**Figure 5:**
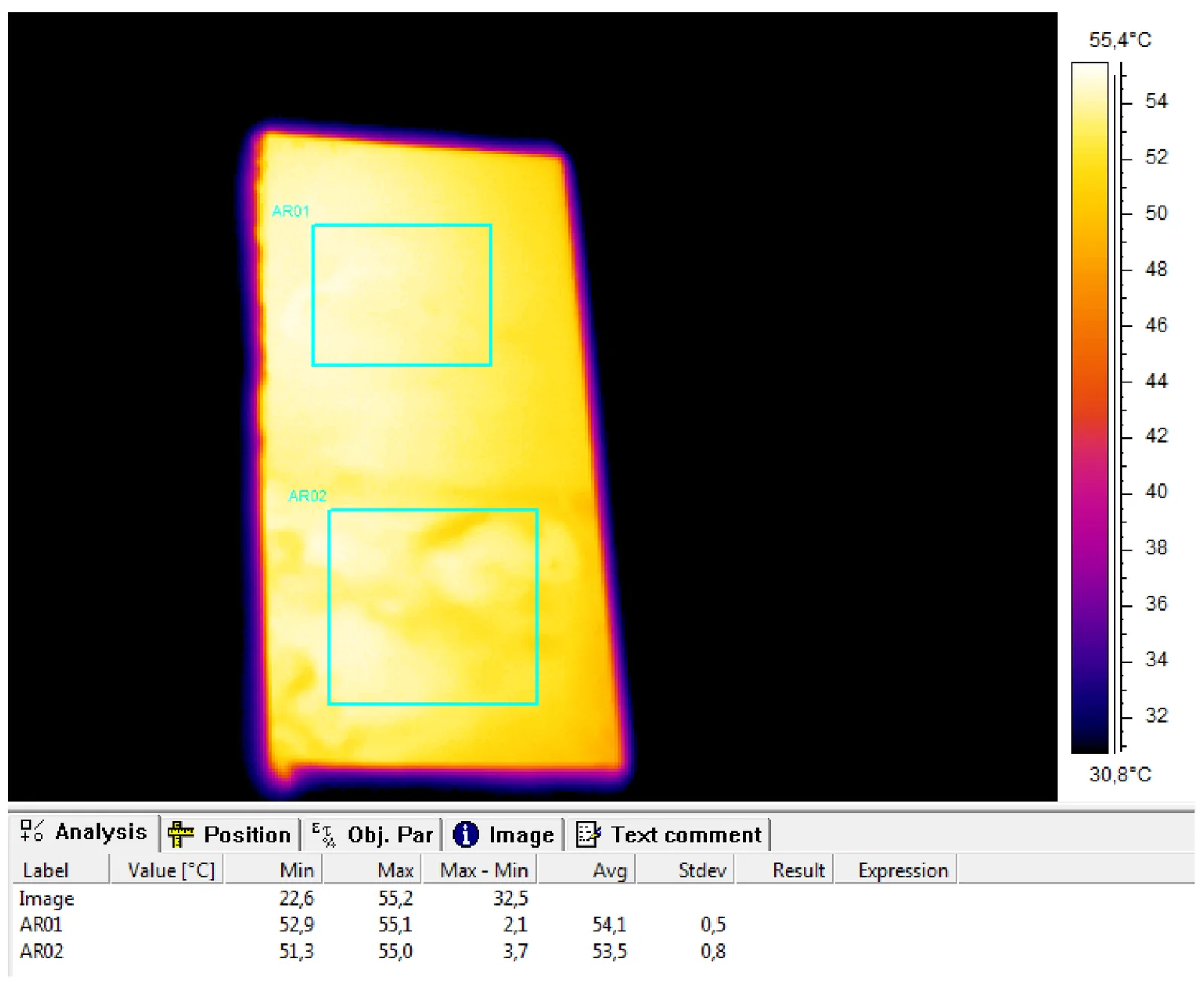
Emissivity measurement. The lower part of the plate was covered with soot. No temperature differences across the plate surface are visible in the thermogram.

We determined the marker surface emissivity using a comparative thermographic method. A portion of the painted aluminium plate was coated with soot, which has a well-established emissivity close to unity (Figure 5). The thermal camera was configured with the soot emissivity, and the apparent temperatures of the coated and uncoated surfaces were compared as the marker was heated above ambient temperature. The measured temperatures were nearly identical, indicating that the painted aluminium surface exhibited an emissivity of approximately 0.95–0.97. Additional observations confirmed that the active surface produced negligible specular reflections compared with the surrounding housing, demonstrating that the marker behaved as an almost diffuse thermal emitter suitable for quantitative thermographic measurements.

**Figure 6:**
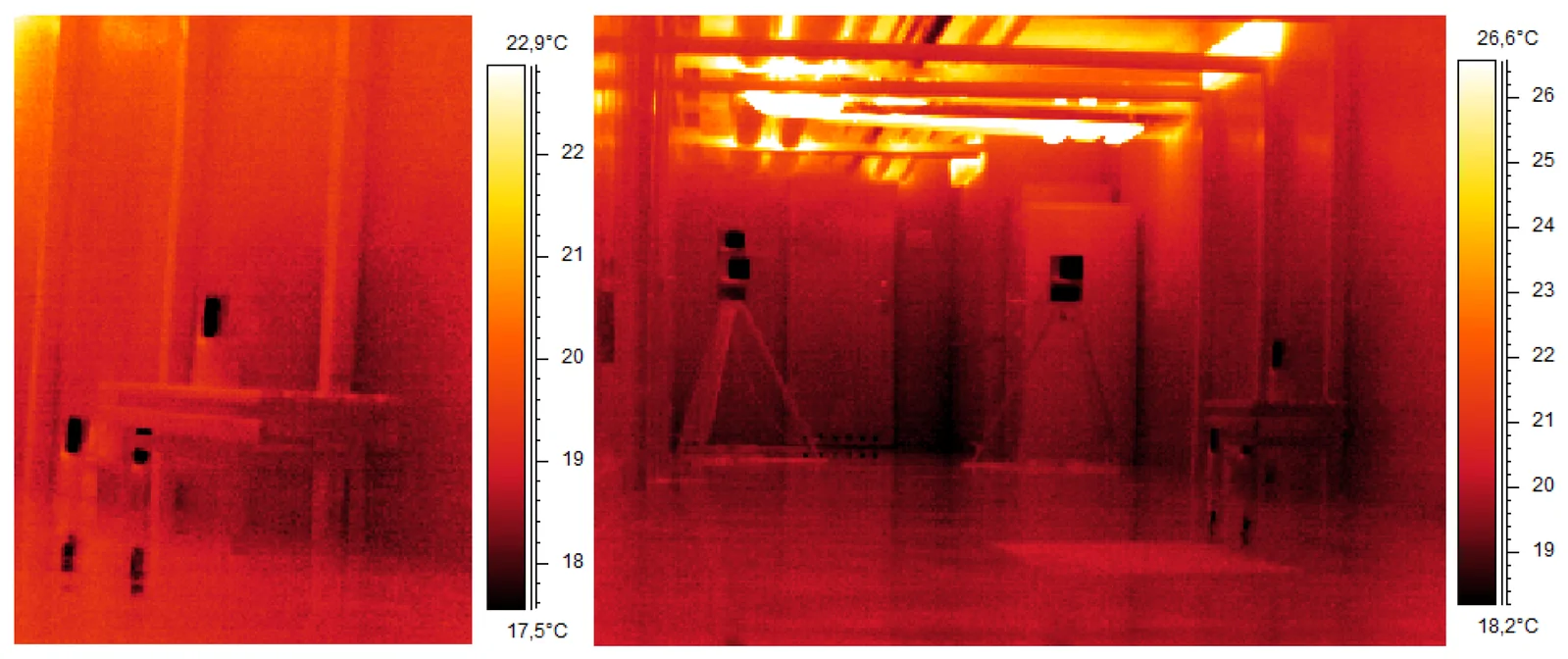
(**Left**): view of the markers at an angle of approximately 45°. (**Right**): view of the markers at an angle of approximately 70° and in a perpendicular orientation.

We evaluated the temperature control system’s performance in two indoor environments representing different atmospheric conditions. In the first experiment, three markers were installed on tripods in a corridor with limited air circulation and operated simultaneously. The marker temperature was set to approximately 15 °C while the ambient temperature remained between 21 and 22 °C. Thermal images were acquired at regular intervals over several hours to evaluate temperature stability and battery performance. All markers maintained a clearly distinguishable thermal signature throughout the experiment, operating continuously for approximately 3 h, with the longest-operating unit remaining functional for nearly 4 h. The average temperature measured from the thermal images differed from the target temperature by approximately 0.5 °C, within the expected measurement uncertainty of the thermal camera (Figure 6).

A second experiment was conducted in a climate-controlled comparator laboratory, where environmental conditions remained highly stable. In this case, the marker temperature was set to approximately 10 °C while the ambient temperature was maintained at approximately 16 °C. Four markers operated successfully for approximately 3 h, maintaining surface temperatures between 10 and 13 °C. The average temperature derived from the thermal images exceeded the programmed temperature by approximately 1.4 °C. This difference was primarily due to condensation forming on the marker surface when its temperature fell below the dew point, altering the apparent thermal emission detected by the infrared camera. In addition, the internal temperature sensor measured the temperature close to the Peltier module rather than directly at the external radiating surface, introducing a small systematic difference between the control temperature and the temperature observed in the thermal images. Consequently, the marker temperature was determined directly from the thermal imagery prior to automated detection instead of relying solely on the internal sensor readings.

We also investigated the influence of imaging geometry. Measurements acquired at distances between 10 and 40 m demonstrated that the markers remained clearly detectable up to approximately 30 m when using a thermal camera with a resolution of 320 × 240 pixels. Tests performed using different viewing angles further showed that the markers could be reliably identified even when the camera optical axis was not perpendicular to the marker surface. Stable detection was achieved at observation angles of approximately 45° and 70°, although increasing distance and viewing angle resulted in a gradual reduction in the apparent marker size and an increase in temperature variability. Additional measurements indicated that local environmental effects, including air circulation, non-uniform solar illumination, and spatial temperature gradients, could influence the apparent surface temperature of individual markers. However, these effects did not compromise marker detection, provided that the expected temperature range was determined from the thermal images before the automated identification procedure.

Overall, the laboratory evaluation demonstrated that the developed active thermal markers provide high emissivity, stable temperature control, and sufficient operational autonomy for practical thermographic surveys. Their stable thermal signature, combined with reliable visibility across a range of acquisition distances and viewing angles, confirmed their suitability as thermal control points for registering thermal imagery with terrestrial laser scanning point clouds. The experiments also highlighted the importance of estimating the actual marker temperature directly from the acquired thermal images, particularly under environmental conditions where condensation or local atmospheric variations may influence the observed radiometric response.

### 4.2. Field Test Evaluation of Active Thermal Markers

The active thermal markers were evaluated in the field on a vegetated slope to assess performance under varying environmental conditions, including high ambient temperatures, morning dew, and solar radiation. Throughout the experiments, the markers maintained stable surface temperatures without noticeable fluctuations, demonstrating reliable operation under outdoor conditions.

The first experiment investigated the maximum temperature difference that could be achieved between the marker surface and its surroundings. With an ambient air temperature of approximately 33 °C and ground surface temperatures reaching 36 °C, the markers were cooled to a minimum surface temperature of approximately 17 °C, corresponding to a temperature contrast of about 14 °C. Although this maximum contrast could only be maintained for approximately 45 min, it demonstrated the cooling capability of the compact Peltier-based system.

A second experiment was conducted to determine the optimum operating temperature that would maximise battery life while maintaining sufficient thermal contrast for reliable detection. Markers were operated at different temperatures between 17 and 30 °C. The results showed that a temperature difference of approximately 6–9 °C below ambient conditions provided the best compromise between visibility and operating time, allowing continuous operation for approximately 3–4 h. Larger temperature differences significantly reduced battery life and promoted condensation on the marker surface, resulting in discrepancies of up to 4 °C between the programmed marker temperature and the temperature measured from thermal imagery. Consequently, the actual marker temperature was estimated directly from the thermograms during image processing.

Finally, we evaluated the markers’ maximum detection distance using a FLIR S60 thermal camera (320 × 240 pixels, 24° field of view). The 100 × 100 mm active Peltier plate remained reliably detectable at distances of approximately 30 m, where it occupied at least nine image pixels and could be clearly distinguished from the surrounding terrain. Although the markers could still be recognised at distances approaching 38 m, the recorded thermal signature increasingly represented the marker housing rather than the active Peltier surface, reducing measurement reliability. To provide more accurate guidance on marker use and expected results, we needed to convert pixel measurements to real distance units. The conversion from pixel error to object-space error was performed using the known coordinates of the camera and the surveyed coordinates of 4–7 reference points visible in each image. The camera position was determined using a GNSS device, while the coordinates of the reference points were known from the survey. These corresponding image points were used to establish the relationship between image coordinates (pixels) and object-space coordinates, allowing the pixel detection error to be expressed as a positional error in the object space. Therefore, the accuracy of the detected markers could be evaluated not only by the number of correctly detected pixels but also by their spatial displacement from the known reference coordinates.

The field experiments confirmed that the developed active thermal markers are suitable for outdoor thermographic surveys. They provide stable temperature control, sufficient battery autonomy for typical measurement campaigns, and reliable visibility over practical acquisition distances. Unlike natural terrain features, whose thermal signatures are often ambiguous or vary with environmental conditions, active markers provide repeatable, easily identifiable control points that facilitate accurate registration of thermal images to three-dimensional point clouds. Future developments will include integrating a ground-temperature sensor, a GNSS receiver for approximate positioning, and improvements to the cooling system and enclosure to further increase operating time and field usability.

## 5. Use of Thermal Markers for Documenting Changes in Materials Deposited in the Coal Spoil Tip

### 5.1. Study Area External Spoil Tip of the Lubelski Węgiel “Bogdanka” S.A. Coal Mine

The study was conducted on the external spoil tip of the Lubelski Węgiel “Bogdanka” S.A. coal mine, located near the village of Puchaczów in the Lublin Coal Basin (eastern Poland). Coal spoil tips are anthropogenic landforms created by the long-term deposition of waste rock generated during underground or open-pit mining operations. In coal mining, the deposited material consists predominantly of claystone, mudstone, sandstone, and shale, along with residual coal fragments and coal dust left after the extraction process. Although the amount of residual coal is relatively small, its gradual oxidation may lead to self-heating and, under favourable conditions, spontaneous combustion within the spoil tip. Such underground fires may remain undetected for years before becoming visible at the surface, producing harmful gases, degrading soil and groundwater quality, and reducing the structure’s geotechnical stability. Consequently, continuous monitoring of spoil tips is considered an important component of mine environmental management [30].

The investigated spoil tip belongs to the Bogdanka hard coal mine, one of the largest coal producers in eastern Poland. The object occupies approximately 88.6 ha, of which about 76 ha constitute the active spoil tip, while the remaining area is used for access roads and drainage infrastructure. The spoil tip reaches heights between 16 and 29 m above the surrounding terrain. The deposited material consists primarily of claystone and mudstone with smaller proportions of shale, sandstone and siderite. The grain-size distribution is dominated by coarse gravel (40–50%), followed by sand (30–40%) and fine-grained silt-sized material. Parts of the south-western slopes have been reclaimed through tree planting to improve slope stability, whereas the central part contains a small water reservoir that is gradually being covered by newly deposited waste material. Previous inspections identified local thermal anomalies associated with self-heating processes, making the Bogdanka spoil tip an appropriate site for evaluating thermal remote sensing methods for early fire detection and environmental monitoring. This site provides a representative example of a modern coal spoil tip, combining relatively simple topography with heterogeneous lithological composition and localised thermal activity. Additionally, the techniques for depositing material have evolved over time from simply depositing waste rock to monitoring particle type and size, and then stabilising slopes with heavy machinery. This causes different parts of the tip to resist water damage in different ways, making the object difficult to monitor [31].

### 5.2. Experiment Setup

**Figure 7:**
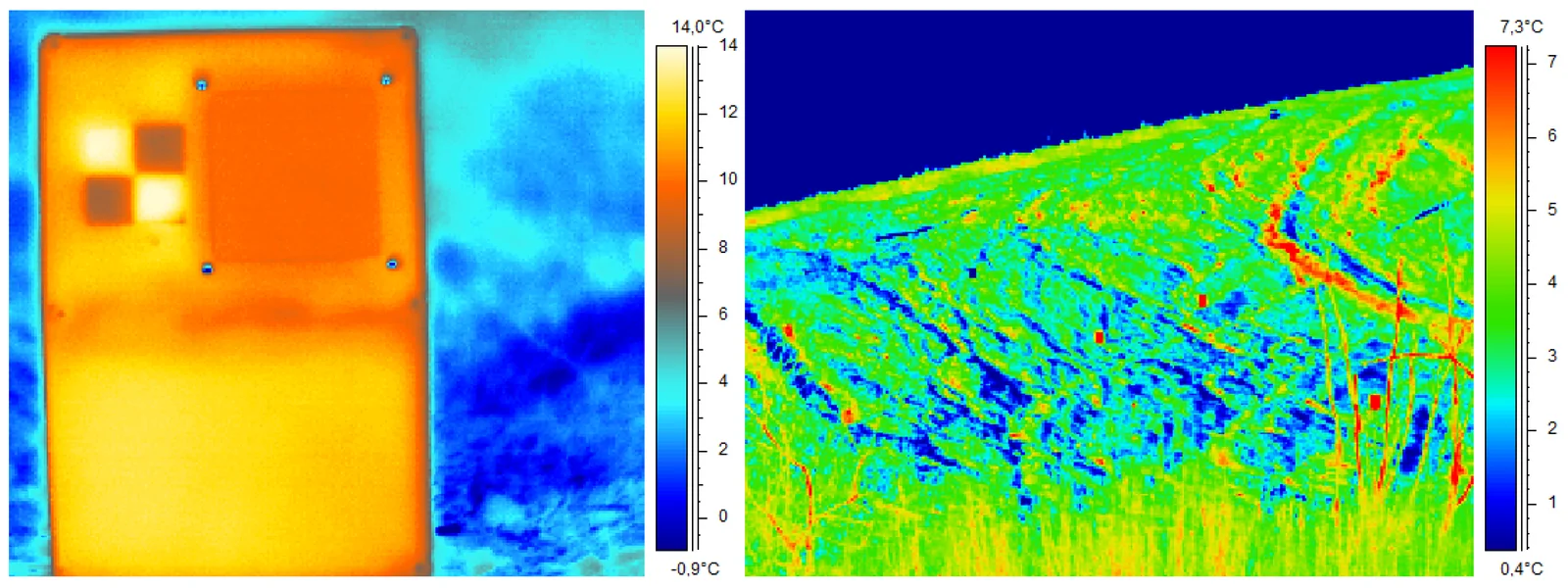
(**Left**): active thermal marker equipped with an attached checkerboard target used as a registration target for the terrestrial laser scanner. (**Right**): distribution of the thermal markers across the spoil tip study area (markers are highlighted in red and enclosed by white circles).

The study was conducted on a section of the external spoil tip of the Lubelski Węgiel Bogdanka coal mine in eastern Poland. Six active thermal markers were distributed across the study area (Figure 7). Each marker was equipped with a checkerboard target attached to its housing, allowing it to serve simultaneously as a thermal control point for infrared image registration and as a reference target for laser scan registration. Additional reference spheres and passive checkerboard targets were deployed throughout the site to improve the geometric registration of the individual scans. Both the coordinates of targets and markers and the position of the IR camera were measured with a GNSS receiver where possible.

The site was surveyed from 10 TLS stations using a FARO Focus3D 120 terrestrial laser scanner [32]. The instrument is a phase-based scanner with a maximum measurement range of 120 m, a ranging accuracy of approximately ±2 mm, and an integrated colour camera for point cloud texturing. It acquires up to 976,000 points per second with a 360° × 305° field of view, making it suitable for high-resolution documentation of complex topographic surfaces. To maximise the spatial coverage of the thermal imagery while minimising the number of active markers required, the markers were relocated four times during the survey. In each measurement cycle, two markers remained fixed while the remaining four were moved to new positions, providing common reference points between consecutive image sets. At each marker configuration, thermal images were acquired from two observation distances, approximately 7 m and 30 m. This acquisition strategy was dictated by the site geometry, as the spoil tip is surrounded by a water-filled drainage ditch, restricting instrument locations to the accessible embankments. To remove distortions from IR images, we removed 10% of the image at each edge. In previous tests, this proved an effective method for removing meaningful distortions without losing large amounts of data. Since this was known before the survey, we had to ensure that all of the markers would be in the part of the image that was not going to be removed.

Six markers were visible in each image during the 30 m survey, and two markers were moved after each image was taken. In the 7 m survey, the aim was to have at least five markers visible in every image. However, because the survey was more complex and the distance shorter, only four markers were visible in some cases. In those cases, for DLT we were aided by a small number of passive markers that had been placed in the areas where we did not have enough active markers to perform DLT with just them. In general, four of the same markers were visible in two adjoining images for the 30 m survey and at least three for the 7 m survey.

We manually extracted the three-dimensional coordinates of the thermal markers from the registered point cloud using point cloud processing software and then used them as control points for the Direct Linear Transformation (DLT). Temperature values were assigned only to points located within the area bounded by the DLT control points, avoiding extrapolation beyond the calibrated image region in the horizontal plane and preventing geometric distortions. In the vertical plane, we also used extrapolated data due to the placement of the markers; however, we took all of the images in a way that 10% of the image (top and bottom) could be removed without losing coverage of the area of interest. Then on points that were overlapping, we checked if the temperature at each of those points was within double the measurement error of this camera. If so, we averaged the temperatures. If not, the point was removed from the point cloud. We usually lost no more than 15% of points. The complete workflow, including field acquisition, scan registration, thermal image processing, and generation of the thermally attributed point cloud, required approximately 6–8 h.

The accuracy of the DLT transformation was assessed using passive reference points with known coordinates on the object. In addition to the thermal markers, the object contained passive reference elements (metal plates) whose relative positions and coordinates were documented on a survey sketch and coordinates measured independently with the use of a GNSS receiver. They were not the same passive markers that we used for DLT in some images where we did not have enough active markers. The passive markers used in DLT were placed separately and only used for DLT; they were not used for any accuracy assessment. The shape and reflectance of passive markers allowed them to be readily identified in both the point cloud and the IR images. After applying the DLT transformation, the GNSS-measured positions of these points were cross-checked against their corresponding locations in the resulting thermal point cloud. Since the reference elements were flat metal plates, they could be clearly identified on the object, allowing us to visually verify whether the transformed IR data correctly covered the 3D surface and whether excessive stretching or displacement was present. In the experiment, three passive reference points were typically visible in the images acquired from 7 m, while four or five were generally visible in the images acquired from 30 m. The accuracy was also cross-checked between adjoining images using the same passive reference points. The quantitative positional error was calculated by comparing the transformed coordinates of the reference points with their known surveyed coordinates. The resulting errors were analysed separately for the 7 m and 30 m acquisition distances using the mean error, RMSE, standard deviation, and maximum error. For the 7 m acquisition distance, the mean positional error was 0.14 m (corresponding to an average of 11 pixels), with an RMSE of 0.21 m, a standard deviation of 0.12 m, and a maximum error of 0.36 m. For the 30 m acquisition distance, the mean positional error was 0.30 m (corresponding to an average of 8 pixels), with an RMSE of 0.34 m, a standard deviation of 0.17 m, and a maximum error of 0.66 m. The maximum error was largely driven by vertical-axis error, where some extrapolation was allowed.

One limitation of the proposed approach concerns areas with complex topography. In deeply incised terrain or on steep slopes, self-occlusions may occur, causing multiple regions of the point cloud to correspond to the same area of a thermal image. Such ambiguities can lead to incorrect temperature assignment. To minimise this effect, we preferentially selected thermal images with the fewest occlusions for processing and visually inspected the resulting thermal distributions to identify duplicated temperature patterns indicative of registration errors. Although effective, this verification remained partially manual and represented one of the most time-consuming stages of the processing workflow.

### 5.3. Discussion

**Figure 8:**
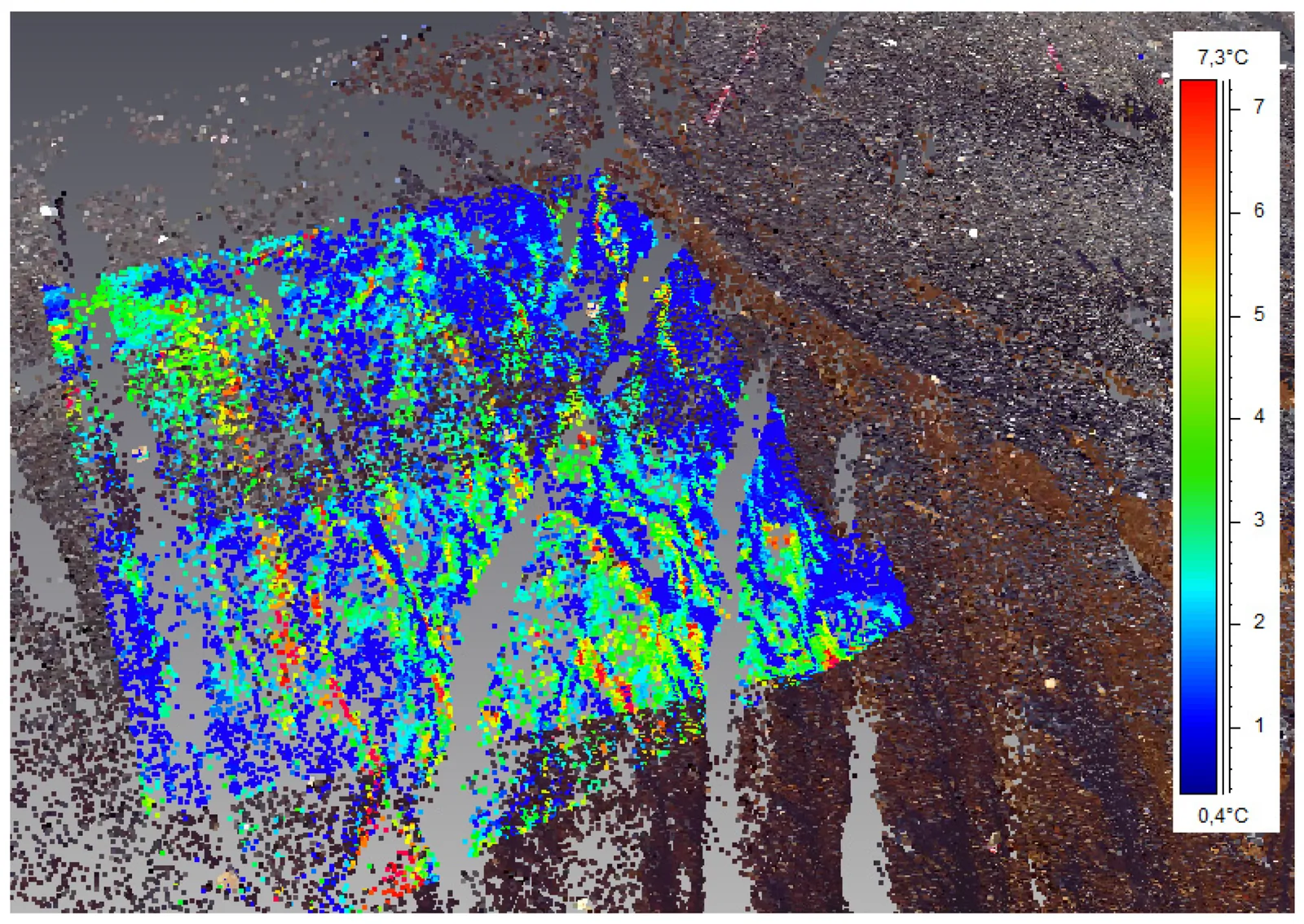
Temperature-coloured point cloud. The temperature values are represented using the midgreen colour scale shown on the right. The remaining points are displayed using their original colours.

As shown in Figure 8, the infrared data are useful not only for identifying potential hotspots and areas at risk of spontaneous combustion, but also for mapping the distribution of different types of material deposited within the spoil tip. The thermal patterns indicate that the deposited material does not follow the original stratification of the spoil tip but is instead redistributed into zones oriented approximately perpendicular to the depositional layers. This redistribution is likely a consequence of successive stabilisation and compaction processes, which exert pressure on the deposited material, causing finer and lighter particles to migrate upwards while larger and denser particles settle towards the base. Such redistribution is particularly important in coal spoil tip monitoring, as it may promote the formation of chimney-like vertical pathways that facilitate air circulation into deeper layers, increasing oxygen availability and potentially initiating or sustaining spontaneous combustion.

The developed workflow successfully integrated terrestrial laser scanning and infrared thermography to generate a three-dimensional thermal digital terrain model of the Bogdanka coal spoil tip. The resulting thermal model enabled the identification and visualisation of local thermal anomalies associated with potential spontaneous-combustion zones, while preserving the three-dimensional morphology of the spoil tip.

## 6. Conclusions

This study shows that actively controlled thermal markers provide an effective solution for geometrically registering terrestrial infrared imagery with three-dimensional topographic data. By maintaining a stable, distinguishable thermal signature, the proposed markers overcome a principal limitation of thermal photogrammetry: the lack of naturally occurring, easily identifiable control points. The developed methodology enables the reliable integration of thermal information with digital terrain models and terrestrial laser scanning point clouds, yielding dense three-dimensional thermal models suitable for quantitative analysis.

The main findings of this research can be summarised as follows:

- Active thermal markers can be automatically detected in thermal imagery due to their stable and controlled surface temperature, allowing extraction of image control points.
- A temperature difference of approximately 6–9 °C relative to the surrounding surface provides a good compromise between marker visibility, battery life, and avoidance of condensation effects under typical field conditions.
- The markers maintained a stable temperature for approximately 3 h, providing sufficient operating time for terrestrial thermal surveying of medium-sized topographic objects.
- The proposed registration workflow based on Direct Linear Transformation (DLT) successfully projects thermal information onto digital terrain models and terrestrial laser scanning point clouds, producing geometrically consistent three-dimensional thermal datasets.
- Combining active thermal markers with terrestrial laser scanning improves the accuracy of control point localisation compared with field surveying alone, while preserving the flexibility of using independent thermal and laser sensors.
- The methodology is applicable to complex natural and anthropogenic landforms, including irregular terrain where conventional photogrammetric targets are difficult or impossible to identify in thermal imagery.
- The resulting thermal point clouds facilitate the interpretation of spatial temperature patterns together with terrain morphology, making them particularly valuable for environmental monitoring, geohazard assessment, and engineering applications.

Experiments conducted at a coal spoil tip demonstrated the practical value of the proposed approach. The integrated thermal point cloud enabled not only the identification of surface thermal anomalies associated with potential spontaneous combustion but also the identification of temperature patterns related to the internal structure and material distribution within the spoil tip. Such information would be difficult to obtain from thermal images or point clouds analysed independently, highlighting the benefits of combining geometric and radiometric information into a single three-dimensional dataset.

Although recent advances in sensor technology have led to commercially available integrated LiDAR–thermal systems, these instruments remain expensive. The proposed marker-based approach offers a cost-effective alternative that can be implemented with standard terrestrial laser scanners and thermal cameras. Furthermore, because the methodology is sensor-independent, it can be adapted to different acquisition platforms and imaging geometries.

Future work should focus on improving the thermal marker design by increasing battery endurance, enhancing thermal stability under highly variable environmental conditions, and integrating GNSS positioning and additional environmental sensors. Further development of automatic registration algorithms and occlusion-handling techniques would also increase the method’s applicability to large-scale surveys and highly complex topographic environments. Overall, the proposed methodology provides a practical framework for generating accurate three-dimensional thermal models of natural and engineered topographic objects, expanding the potential of infrared remote sensing for quantitative spatial analysis.

## Data Availability

All data, including MATLAB scripts, can be made available on request.

## Abbreviations

The following abbreviations are used in this manuscript:

DLT	Direct Linear Transformation
GCP	Ground Control Point
GNSS	Global Navigation Satellite System
IR	Infrared
LiDAR	Light Detection and Ranging
RGB	Red Green Blue
TLS	Terrestrial Laser Scanner
